# Early Postoperative Complications after Decompressive Craniectomy for Pediatric Severe Traumatic Brain Injury

**DOI:** 10.21203/rs.3.rs-10102100/v1

**Published:** 2026-07-01

**Authors:** Zhanna B. Semenova, Denis E. Bragin, Evgenii A. Rogozhin, Vladimir I. Lukyanov, Semen V. Meshcheryakov

**Affiliations:** 1 Clinical and Research Institute of Emergency Pediatric Surgery and Trauma, Moscow, Russia; 2 Lovelace Biomedical Research Institute, Albuquerque, NM, USA; 3 Department of Neurology, School of Medicine, University of New Mexico, Albuquerque, NM, USA; 4 Department of Cell and Molecular Physiology, New York Medical College, Valhalla, NY, USA

**Keywords:** Pediatric Neurocritical Care, Traumatic Brain Injury, Decompressive Craniectomy, Intracranial Pressure, Postoperative Complications

## Abstract

**Background:**

Decompressive craniectomy (DC) is used to treat refractory intracranial hypertension after pediatric severe traumatic brain injury (sTBI), but early postoperative neurosurgical complications and their predictors—particularly intracranial pressure (ICP) burden—remain inconsistently characterized.

**Methods:**

We performed a retrospective cohort study of consecutive pediatric patients (<18 years) with sTBI (Glasgow Coma Scale [GCS] 3–8) who underwent DC at a single high-volume trauma center. Early postoperative neurosurgical complications were defined a priori as events occurring within 7 days after DC or before hospital discharge: secondary intracranial hemorrhage, central nervous system (CNS) infection, wound dehiscence, and cerebrospinal fluid (CSF) leak. Associations with injury severity, imaging, and ICP variables (mean intraoperative ICP; cumulative duration of ICP >20 mmHg before DC) were evaluated.

**Results:**

Eighty-three children were included. At least one early neurosurgical complication occurred in 33 of 83 patients (39.8%). Secondary intracranial hemorrhage occurred in 29/83 (34.9%), CNS infection in 7/83 (8.4%), wound dehiscence in 6/83 (7.2%), and CSF leak in 4/83 (4.8%). Patients with complications had significantly higher intraoperative ICP (40.5 ± 13.5 vs 28.6 ± 4.3 mmHg; p = 0.0058) and longer pre-DC duration of ICP >20 mmHg (4.1 ± 5.9 vs 1.6 ± 2.5 hours; p = 0.0296). At 6 months, 25/83 (30.1%) had died; among survivors, 28/58 (48.3%) achieved a favorable outcome (GOS 4–5), 12% in 1 group (without complications).

**Conclusions:**

Early postoperative complications after pediatric DC were common and predominantly hemorrhagic. A greater ICP burden before and during DC was associated with these events. The co-occurrence of CSF leak and CNS infection highlights the importance of dural closure strategies that minimize CSF egress and the need for vigilant postoperative surveillance.

## Introduction

Traumatic brain injury (TBI) remains a leading cause of death and long-term disability in children, and severe TBI (sTBI) carries a high risk of secondary brain injury mediated by intracranial hypertension and systemic insults [1,2]. Contemporary guidelines emphasize aggressive prevention and treatment of secondary injury, including targeted management of elevated intracranial pressure (ICP) [3,4]. When intracranial hypertension remains refractory to optimized medical therapy, decompressive craniectomy (DC) is commonly used as a rescue intervention to reduce ICP and prevent herniation [3–5].

Although DC can effectively lower ICP, its risk–benefit profile is complex. Randomized trials in adults (DECRA and RESCUEicp) demonstrated robust ICP reduction but also highlighted substantial postoperative morbidity and nuanced functional outcomes [5,6]. Reported neurosurgical complications include hemorrhagic progression of contusions, new extra-axial hematomas, infection, cerebrospinal fluid (CSF) leak, and wound breakdown [7–10]. Hemorrhagic progression after decompression has been linked to abrupt pressure shifts and altered cerebral hemodynamics, conceptually including loss of “tamponade” across injured microvasculature in contused tissue [7–9].

In pediatrics, the evidence base is less mature. Most reports are small single-center series or heterogeneous multicenter datasets with variable surgical indications, monitoring practices, operative techniques, and definitions of complications, contributing to wide variation in reported complication rates [1,2,11–13]. Systematic reviews underscore persistent uncertainty about the incidence and predictors of complications after pediatric DC, as well as the limited availability of high-quality early postoperative data [11]. Pediatric-specific anatomical and physiological features (including higher brain water content and distinct compliance characteristics) may further modulate the risk of complications compared with adults [3].

Accordingly, the primary objective of this study was to quantify the incidence and spectrum of early postoperative neurosurgical complications after DC in children with sTBI treated at a single high-volume pediatric trauma center. The secondary objective was to identify clinical, radiological, and ICP-related factors associated with these complications, with particular emphasis on mean intraoperative ICP and the cumulative preoperative duration of ICP >20 mmHg [3,4]. We hypothesized that greater preoperative ICP burden and higher intraoperative ICP would be associated with a higher risk of early postoperative complications, particularly secondary intracranial hemorrhage.

## Methods

### Study Design and Setting

We conducted a retrospective cohort study at the Clinical and Research Institute of Emergency Pediatric Surgery and Trauma (Moscow, Russian Federation). The study adhered to the Strengthening the Reporting of Observational Studies in Epidemiology (STROBE) guidelines for observational studies [14] and followed the ethical principles of the Declaration of Helsinki [15].

The institutional review board approved the study (protocol #027–24). Given the retrospective design and use of de-identified data, the requirement for written informed consent was waived in accordance with institutional and national regulations. Medical records were reviewed for pediatric patients with severe traumatic brain injury (sTBI) who underwent decompressive craniectomy (DC) for refractory intracranial hypertension between 2007 and 2023.

### Participants

Inclusion criteria were:

- Were younger than 18 years at the time of injury;
- Had sTBI, defined as a Glasgow Coma Scale (GCS) score of 3–8 on admission [16]; and
- Underwent DC during the index hospitalization for refractory intracranial hypertension and/or mass-effect–related herniation unresponsive to maximal medical therapy [3,4].

Exclusion criteria were:

- Mild or moderate TBI (GCS >8);
- No DC performed during the index hospitalization;
- Non-traumatic etiologies (e.g., stroke, infection);
- Incomplete medical records precluding assessment of complications or ICP variables; or
- Death before any postoperative neuroimaging or clinical documentation of early postoperative events.

A total of 83 consecutive patients met the inclusion criteria and were included in the analysis. The cohort comprised 54 boys (65.1%) and 29 girls (34.9%), with a mean age of 10.4 ± 4.6 years. The main mechanisms of injury were road traffic accidents (46 children, 55.4%) and falls from height (16 children, 19.3%), with other causes accounting for the remaining 25.3% (21 children). Combined injuries (polytrauma) were present in 65 patients (78.3%), as defined by an Injury Severity Score (ISS) >15 [17].

### Data Collection

Data were abstracted from electronic medical records, prehospital documentation, and operative and radiology reports using a standardized data collection form. The following variables were collected:

- Demographics: age, sex
- Neurologic status: admission GCS [16]
- Pupillary reactivity: both reactive, unilateral non-reactive, bilateral non-reactive
- Prehospital secondary insults: hypotension (systolic BP <90 mmHg or age-adjusted equivalent) and hypoxia (SpO_2_ <90%)
- Trauma severity: Injury Severity Score (ISS) [17]
- Neuroimaging: initial head CT classified using the Marshall CT classification [18]; when available, brain MRI graded using the Firsching classification [19]
- ICP-related variables: presence/absence of invasive monitoring; mean intraoperative ICP; cumulative duration of ICP >20 mmHg before DC (see below)
- Surgical characteristics: DC approach (bifrontal, unilateral, bilateral), primary vs secondary DC (definitions below), dural opening/closure strategy and use of dural substitute (as documented)
- Outcomes: early postoperative neurosurgical complications and Glasgow Outcome Scale (GOS) at 1 and 6 months [20]

### Intracranial Pressure Monitoring and Preoperative Management

Continuous ICP monitoring was established in 69 of 83 patients (83%) using intraventricular catheters or intraparenchymal strain-gauge sensors (Codman), in accordance with guidelines (2019). In 14 patients, monitoring was not performed due to rapid neurological deterioration (dislocation syndrome). ICP values were documented for all patients who had ICP monitoring

Preoperative management followed contemporary severe TBI recommendations, including sedation/analgesia, head elevation, normothermia, normoventilation with brief hyperventilation reserved for impending herniation, hyperosmolar therapy, and hemodynamic support to preserve age-appropriate cerebral perfusion pressure [3,4].

### Indications for DC

- Refractory intracranial hypertension: sustained ICP >20 mmHg despite optimized medical therapy [3,4] and/or
- Clinical deterioration with radiologic mass effect/herniation consistent with a dislocation syndrome

### Pre-specified ICP Exposure Metrics

Two ICP metrics were defined a priori:

1. Mean intraoperative ICP: the average ICP value documented during DC (from the anesthetic record).
2. Pre-DC ICP burden: cumulative duration (hours) of ICP >20 mmHg from initiation of invasive monitoring to skin incision for DC.

### Surgical Procedures

The decision to DC was made by the neurosurgeon and intensive care specialist [5,9]. Bifrontal DC was performed in 37 patients (44.6%), unilateral hemicraniectomy in 44 (53.0%), and bilateral DC in 2 (2.4%). In our series, ICP monitoring was carried out in 69 of 83 patients (83.1%), and primary DC in 14 of 83 patients (16.9%). Standard postoperative care included intensive care neuromonitoring.

### Outcome Definitions

Early postoperative complications were defined a priori as adverse events occurring from the end of DC until hospital discharge or within 7 days postoperatively, whichever occurred first, and directly related to the central nervous system (CNS) or surgical site. The primary outcome was the occurrence of at least one of the following:

- Secondary intracranial hemorrhage, including:
  - Expansion of pre-existing intracerebral hemorrhagic contusions;
  - New ipsilateral hematomas (epidural or subdural) in the decompressed hemisphere;
  - New contralateral intracranial hematomas.
- Central nervous system infection, defined as meningitis or ventriculitis based on clinical signs, cerebrospinal fluid (CSF) findings, and/or imaging, as judged by the treating team [21].
- Postoperative suture dehiscence is defined as partial or complete wound breakdown requiring additional wound care, resuturing, or surgical revision.
- CSF leak, defined as clinically apparent wound liquorrhea or subgaleal CSF collection at the incision site, confirmed by examination and/or imaging.

Complications involving other organs or systems (e.g., sepsis, fat embolism, pulmonary edema) were recorded and analyzed descriptively but were not included in the primary definition of “early postoperative complication.” Reoperation for treatment of secondary intracranial hemorrhage was recorded, including the indication and timing (evacuation of a new ipsilateral subdural hematoma on postoperative day 5).

### Functional Outcomes

Functional outcomes were assessed using the Glasgow Outcome Scale (GOS) at 1 and 6 months after DC [20]. Outcomes were categorized as unfavorable (GOS 1–3) or favorable (GOS 4–5) for secondary analyses. Patients were stratified into two groups for comparative analysis: those without early postoperative complications and those with at least one early postoperative complication.

### Statistical Analysis

Statistical analyses were performed using MS Excel 2007 and Statistica v.6, SPSS v.10, MedCalc v.13. Continuous variables were assessed for normality using the Shapiro–Wilk test. Normally distributed variables are presented as mean ± standard deviation (SD), while non-normally distributed variables are presented as median (interquartile range [IQR]). Categorical variables are summarized as counts and percentages.

Between-group comparisons (no complications vs. ≥1 early complication) were conducted as follows:

- Student’s t-test or Mann–Whitney U test for continuous variables, as appropriate;
- Chi-square test or Fisher’s exact test for categorical variables, as appropriate.

Variables with p < 0.10 in univariable analyses, along with clinically relevant variables selected a priori (e.g., age, admission GCS, ISS, CT Marshall classification, presence of diffuse cerebral edema, intraoperative ICP, duration of ICP >20 mmHg), were entered into a multivariable logistic regression model to identify independent predictors of early postoperative complications. Results are reported as odds ratios (ORs) with 95% confidence intervals (CIs). Model fit was evaluated using the Hosmer–Lemeshow goodness-of-fit test, and multicollinearity was assessed using variance inflation factors, in line with standard recommendations for logistic regression modeling.

A two-sided p-value < 0.05 was considered statistically significant.

## Results

### Patient Characteristics and Baseline Injury Severity

Eighty-three children with severe traumatic brain injury (sTBI) underwent decompressive craniectomy (DC) and were included in the analysis. The cohort comprised 54 boys (65.1%) and 29 girls (34.9%), with a mean age of 10.4 ± 4.6 years. The mean admission Glasgow Coma Scale (GCS) score was 6.4 ± 1.4 (range 3–8), confirming severe TBI in all patients [16]. The main mechanisms of injury were road traffic accidents (46/83, 55.4%) and falls from height (16/83, 19.3%); other mechanisms accounted for 21/83 (25.3%). Combined injuries (polytrauma) were present in 65 patients (78.3%), defined as Injury Severity Score (ISS) > 15; mean ISS for the overall cohort was 29.74 ± 7.45 [17].

Initial head computed tomography (CT) patterns were classified using the Marshall CT classification: type 2 in 15 patients (18.1%), type 3 in 45 (54.2%), and type 4 in 23 (27.7%) (Figure 1) [18]. When available, magnetic resonance imaging (MRI) findings were graded using the Firsching classification [19]. Continuous intracranial pressure (ICP) monitoring was established in 69/83 patients (83.1%). Episodes of prehospital hypotension and hypoxia were documented in 20% and 17% of patients, respectively.

### Operative Characteristics

Surgical approach was determined by lesion distribution and clinical judgment: bifrontal DC was performed in 37 patients (44.6%), unilateral hemicraniectomy in 44 (53.0%), and bilateral DC in 2 (2.4%). Secondary DC was performed in 69 patients (83.1%), and primary DC in 14 patients (16.9%).

### Incidence and Spectrum of Early Postoperative Complications

Early postoperative complications, defined as events occurring within 7 days after DC or before hospital discharge, developed in 33 of 83 patients (39.8%; 95% confidence interval [CI] 29.2–50.4%). Because complication categories were not mutually exclusive, some patients experienced >1 complication. Across the cohort, the total number of complication-category events was 46 (29 hemorrhagic + 7 infectious + 6 dehiscence + 4 CSF leak), occurring in 33 patients. The distribution of complication types is summarized in Table 1.

Secondary intracranial hemorrhage represented the dominant complication, occurring in 29 patients (34.9%; 95% CI 24.7–45.1%). Central nervous system (CNS) infections (meningitis or ventriculitis) were diagnosed in 7 patients (8.4%; 95% CI 2.4–14.4%) [21]. Wound-related complications included postoperative suture dehiscence in 6 patients (7.2%; 95% CI 1.6–12.8%) and clinically evident cerebrospinal fluid (CSF) leak (wound liquorrhea) in 4 patients (4.8%; 95% CI 0.2–9.4%).

Although early neurosurgical complications were the primary focus of this analysis, systemic complications involving other organs and systems (e.g., sepsis, fat embolism, pulmonary edema) also occurred and contributed to death in 5 patients. These systemic events were not classified as early postoperative neurosurgical complications in the primary outcome definition but are clinically relevant when interpreting overall morbidity and mortality in this high-risk cohort.

### Characteristics of Secondary Intracranial Hemorrhages

Secondary intracranial hemorrhages were the most frequent early postoperative complication, observed in 29 of the 33 children with ≥1 complication (87.9%). The observed hemorrhage rate in this series exceeded that reported in a recent pediatric DC cohort [22], underscoring the particular vulnerability of this population to hemorrhagic progression after decompressive surgery.

**Figure 2.**
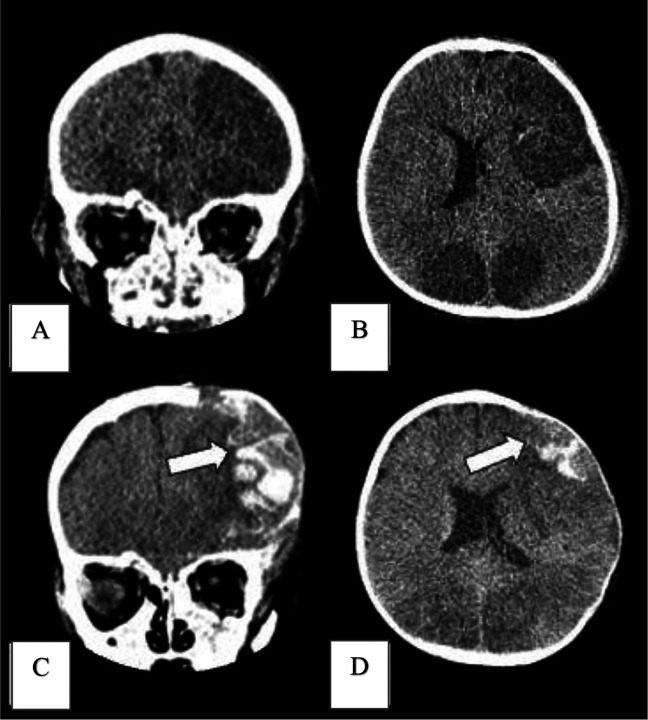
Computed tomograms of the patient’s brain at admission, A (frontal section), B (axial section). Computed tomograms of the patient’s brain after DC (frontal section), G (axial section). The arrow shows an increase in the hemorrhagic focus of the injury in the early postoperative period.

The most common hemorrhagic pattern was expansion of pre-existing intracerebral hemorrhagic contusions, diagnosed in 24 of 29 patients with secondary hemorrhage (82.8% of hemorrhagic complications). In these cases, follow-up CT imaging demonstrated enlargement of contusional foci, often with increased surrounding edema and mass effect compared with preoperative scans (Figure 2).

**Figure 3.**
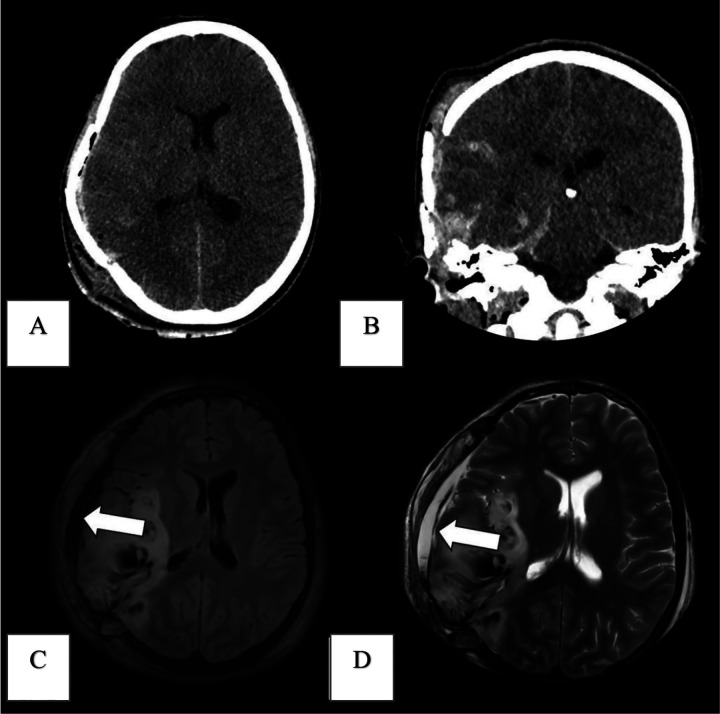
Computed tomograms of the patient’s brain at admission, A (axial section), B (frontal section). MRI of the patient after DC. C (axial section T1 mode), D (axial section T2 mode). The arrow shows the development of an acute subdural hematoma in the early postoperative period.

New ipsilateral hematomas developed in 4 patients (13.8% of hemorrhagic complications): two acute epidural hematomas and two acute subdural hematomas in the decompressed hemisphere. In one child with an ipsilateral acute subdural hematoma, mass effect progressed over the first postoperative days, necessitating repeat surgical evacuation on postoperative day 5 (Figure 3). The other ipsilateral hematomas were managed conservatively based on hematoma size, stability on serial imaging, and clinical status.

**Figure 4.**
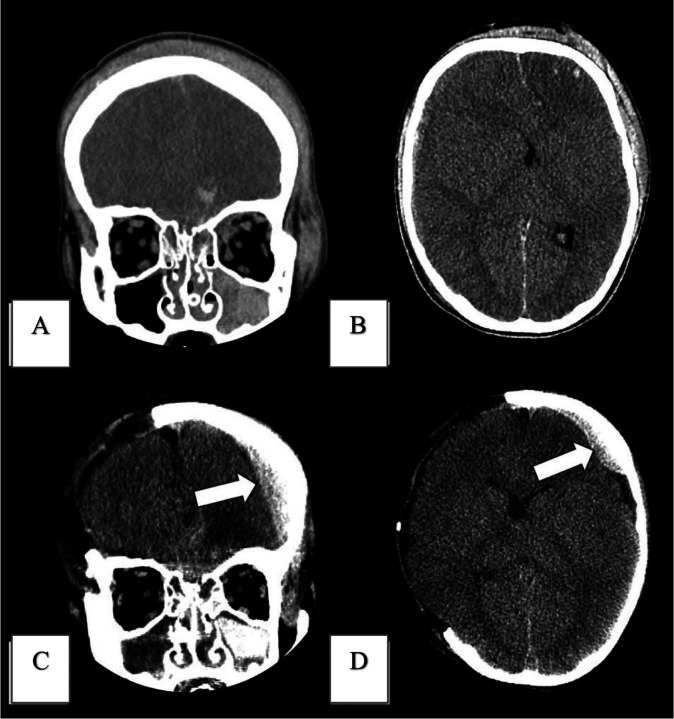
Computed tomograms of the patient’s brain at admission, A (frontal section), B (axial section). Computed tomograms of the patient’s brain after DC. B (frontal section), D (axial section). The arrow shows the development of an acute subdural hematoma in the early postoperative period.

**Figure 5.**
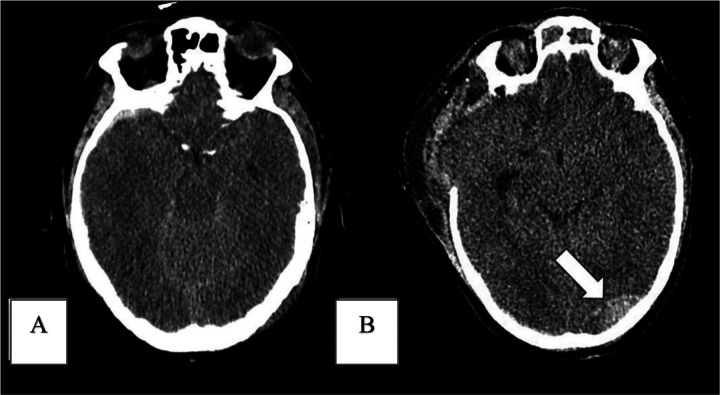
Computed tomograms of the patient’s brain at admission, A (axial section), B (axial section) - after DC. The arrow shows the development of an acute epidural hematoma in the early postoperative period.

Contralateral hematomas occurred in 2 patients (6.9% of hemorrhagic complications). In both cases, the hematoma volume on CT was <20 mL, and no surgical evacuation was required (Figures 4 and 5). These lesions were monitored with serial neuroimaging and clinical assessment and remained radiologically and clinically stable.

Overall, hemorrhagic complications were characterized predominantly by contusion expansion, with a smaller subset of children developing new ipsilateral or contralateral extra-axial hematomas. Only one child (1.2% of the total cohort) required early repeat neurosurgical intervention specifically for a secondary intracranial hemorrhage.

### Infectious Complications, CSF Leak, and Wound Dehiscence

Central nervous system infections (meningitis or ventriculitis) were documented in 7 patients (8.4%). These infectious complications typically occurred in the context of complex wound-healing and dural-closure challenges. In 4 of the 7 infected patients, primary duraplasty could not be completed in a fully watertight fashion due to marked intraoperative brain swelling and prolapse of edematous parenchyma through the dural defect. All 4 of these patients subsequently developed postoperative wound CSF leakage, suggesting a close mechanistic association between non-hermetic dural closure, CSF leak, and CNS infection.

**Figure 6.**
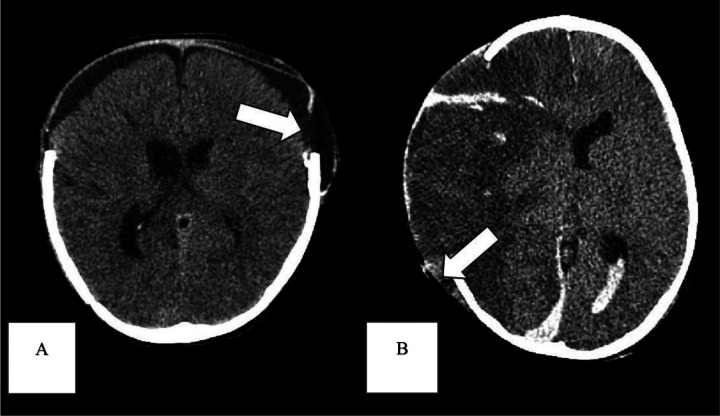
Computed tomograms of the patient’s brain at admission A (axial section), B (axial section) - after DC. The arrow shows the development of an acute epidural hematoma in the early postoperative period.

Clinically evident CSF leak (wound liquorrhea) was observed in 4 patients (4.8%). CSF leakage typically manifested within the first 2 postoperative days as clear fluid drainage along the incision line or collection beneath the scalp flap. In all affected patients, CSF leak was associated with substantial intraoperative brain edema and technical difficulty achieving watertight dural closure (Figure 6). CSF leakage often co-occurred with other wound-related complications and was a prominent feature in the subgroup of patients who developed meningitis or ventriculitis.

Postoperative suture dehiscence occurred in 6 patients (7.2%). Dehiscence ranged from partial separation of the skin edges with localized breakdown to more extensive wound disruption. Persistent CSF leakage, excessive tension on the scalp flap, and postoperative swelling of underlying tissues appeared to be the main contributing factors. This clustering of wound complications is consistent with observations in pediatric DC series that emphasize the relationship among swelling, closure difficulty, CSF egress, wound failure, and infection risk [23].

### Risk Factors for Early Postoperative Complications

To identify predictors of early complications, patients were stratified into two groups: Group 1 (no complications, n = 50) and Group 2 (≥1 complication, n = 33). Baseline characteristics and comparative analyses are summarized in Table 2.

Baseline injury severity: Patients in Group 2 had significantly lower mean admission GCS (4.6 ± 1.8 vs 7.3 ± 1.0 in Group 1; p < 0.001) [16], higher mean Marshall CT score (3.2 ± 0.5 vs 2.8 ± 0.7; p = 0.01) [18], and higher mean Firsching classification grade (2.0 ± 0.7 vs 1.4 ± 0.6; p = 0.002) [19], indicating more severe primary brain injury and greater mass effect.

Prehospital secondary insults: Prehospital hypotension and hypoxia were more frequent in Group 2 (hypotension: 7/33 [21.2%] vs 4/50 [8.0%], p = 0.04; hypoxia: 6/33 [18.2%] vs 3/50 [6.0%], p = 0.03).

**Figure 7.**
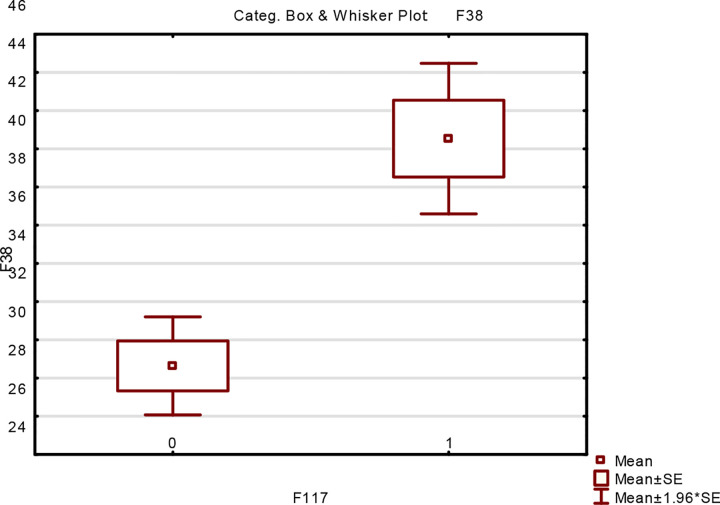
The average value of ICP in patients of groups 1 (0) and 2 (1). Mean is the average value, Mean±SE is the error of the average value, and Mean±1.96*SE is the 95% confidence interval of the mean value.

**Figure 8.**
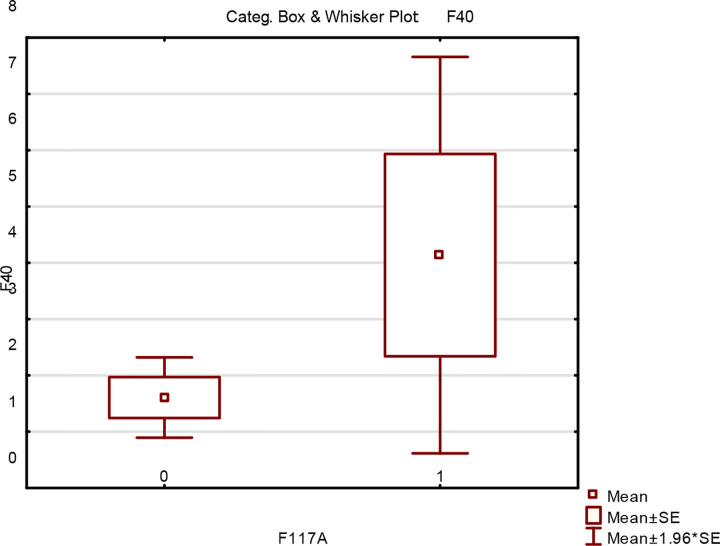
The average duration of ICP above 20 mmHg to DC is higher in patients in groups 1 (0) and 2 (1). Mean is the average value, Mean±SE is the error of the average value, and Mean±1.96*SE is the 95% confidence interval of the mean value.

Intracranial pressure burden: A comparative multivariable analysis between the two groups showed statistically significant differences in the magnitude and duration of ICP before DC. The average ICP at the time of surgery in patients without complications (Group 1) was lower, at 28.6 ± 4.3 mmHg, whereas in the second group (Group 2) it averaged 40.5 ± 13.5 mmHg (p = 0.00582). The distribution is shown in Figure 7. Similarly, the average duration of ICP above 20 mmHg before DC in patients in Group 1 was 1.6 ± 2.5 hours. In patients in Group 2, the average duration of ICP was approximately twice that of Group 1, at 4.1 ± 5.9 hours (p = 0.02959). The distribution is shown in Figure 8.

Systemic trauma severity: Higher systemic trauma burden was also associated with complications. Mean ISS was 35.8 ± 7.1 in Group 2 compared with 25.4 ± 6.2 in Group 1 (p < 0.001) [17].

In multivariable logistic regression, three independent predictors of early postoperative neurosurgical complications were identified:

- Sustained intraoperative ICP >40 mmHg (odds ratio [OR] 3.2; 95% CI 1.5–6.8; p = 0.003)
- ISS >25 (OR 2.1; 95% CI 1.1–4.0; p = 0.02) [17]
- Diffuse cerebral edema on initial CT (OR 2.5; 95% CI 1.2–5.1; p = 0.01) [18]

These predictors align with factors reported in prior DC complication literature [7–9].

### Reoperations

Only one patient (1.2%) required repeat surgery in the early postoperative period. This child developed a new ipsilateral acute subdural hematoma on postoperative day 5, which was treated by surgical evacuation.

### Functional Outcomes

Functional outcomes were assessed using the Glasgow Outcome Scale (GOS) [20]. At 1 month after DC, among all 83 patients:

- Unfavorable outcome (GOS 1–3) was observed in 25 patients (30.1%);
- Severe disability (GOS 3) was recorded in 44 patients (53.0%);
- Favorable outcome (GOS 4–5) was present in 14 patients (16.9%).

At 6 months, 25 patients (30.1%) had died, leaving 58 survivors. Among survivors:

- Severe disability (GOS 3) was present in 30 patients (51.7%);
- Favorable outcome (GOS 4–5) was observed in 28 patients (48.3%).

These findings indicate a degree of functional recovery over time in a substantial proportion of children despite severe initial injury.

## Discussion

### Early Postoperative Complications After Pediatric Decompressive Craniectomy

Decompressive craniectomy (DC) remains a cornerstone rescue intervention for refractory intracranial hypertension after pediatric severe traumatic brain injury (sTBI), yet the early postoperative period is vulnerable to complications that can exacerbate secondary brain injury. In this cohort of 83 children, 39.8% (33/83) experienced at least one early postoperative neurosurgical complication. Secondary intracranial hemorrhage predominated (34.9%, 29/83), most commonly as expansion of pre-existing hemorrhagic contusions (82.8% of hemorrhagic events), while CNS infection occurred in 8.4% (7/83), suture dehiscence in 7.2% (6/83), and CSF leak in 4.8% (4/83). Complication categories were not mutually exclusive, yielding 46 discrete complication events across the 33 affected patients.

### Limitations of Adult Data and the Need for Pediatric-Specific Evidence

The conceptual framework for DC-related complications is largely derived from adult series and randomized trials (DECRA and RESCUEicp), which established that DC effectively lowers intracranial pressure (ICP) but carries a nuanced risk–benefit profile and substantial postoperative morbidity [5,6]. However, pediatric patients differ fundamentally from adults in cranial biomechanics, brain water content, edema kinetics, and injury patterns [11,12,13]. Children exhibit age-dependent cranial compliance, greater brain hydrophilicity, and a propensity for rapid, diffuse swelling and systemic responses that reshape the risk and timing of complications. Systematic reviews of pediatric DC underscore the heterogeneity of reported complication rates, attributable to small cohorts, variable operative timing and technique, and differences in institutional neurocritical care infrastructure [11]. Pediatric-specific data are therefore indispensable for defining dominant complications and identifying actionable upstream factors in this population [11].

### Hemorrhagic Complications: Predominance, Mechanisms, and Clinical Impact

Hemorrhagic complications constituted the principal early postoperative event in our cohort, consistent with adult and pediatric observational studies describing contusion expansion and new hematoma formation after decompression [7,8,9,10]. Mechanistically, the abrupt restoration of intracranial compliance after DC can eliminate local tamponade, thereby producing regional hyperemia, hyperperfusion, and microvascular destabilization in injured tissue [8,9]. These processes are plausible in children but appear to be amplified by pediatric physiology: higher brain water content, brisk edema formation, venous congestion, and diffuse axonal injury patterns increase tissue strain and hemorrhagic susceptibility when decompression occurs at extreme ICP [12,13,24]. Our data adds granularity by demonstrating that hemorrhagic progression was overwhelmingly contusion expansion rather than de novo extra-axial hematomas. Although the radiographic incidence was high, the reoperation rate remained low (1.2%), indicating that many lesions were managed nonoperatively with intensified neurocritical care. Nevertheless, even nonoperative hemorrhagic expansion can prolong mass effect, extend ICP-directed therapy, delay sedation/ventilation weaning, and heighten exposure to secondary insults—factors particularly detrimental to developmental recovery trajectories in children [25,24].

### ICP Burden as an Upstream Driver of Early Complications

A central pediatric contribution of this study is the identification of ICP burden as an upstream driver of early complications. Children who developed complications exhibited significantly higher mean intraoperative ICP and longer cumulative duration of ICP >20 mmHg prior to DC; in multivariable analysis, sustained intraoperative ICP >40 mmHg emerged as an independent predictor. These observations align with Brain Trauma Foundation pediatric guidelines that define sustained intracranial hypertension as a key escalation threshold and underscore its strong association with adverse outcomes [3]. Pediatric physiology compresses the ICP–swelling timeline relative to adults, rendering the surgical field more hostile (tense parenchyma, difficult hemostasis, heightened postoperative swelling risk) [12]. Our findings, therefore, contextualize adult RCT data: while DECRA and RESCUEicp established ICP control in adults [5,6], they do not delineate pediatric “safe windows” or the accelerated cascade of complications observed when surgery is performed after prolonged, refractory ICP elevation.

### Infectious and Wound-Related Complications: CSF Leak as a Pivotal Node

Infectious and wound-related complications, though less frequent than hemorrhage, carried important clinical weight and clustered tightly. All four cases of CSF leak occurred within the infected subgroup, supporting a mechanistic pathway in which CSF egress serves as a conduit for microbial ingress and promotes wound maceration. CSF leak and infection were frequently linked to intraoperative situations in which severe swelling and parenchymal prolapse precluded fully watertight duraplasty, a challenge intrinsic to pediatric DC given the hydrophilic, rapidly edematous injured brain. This tension between maximal decompression and hermetic closure is amplified in children relative to adults and explains the observed wound dehiscence–CSF leak–infection triad [23,26]. When watertight reconstruction is not achievable, heightened postoperative wound surveillance and prompt escalation of CSF diversion or re-exploration become critical priorities in neurocritical care. Diagnostic and therapeutic management of suspected postoperative ventriculitis or meningitis should follow established Infectious Diseases Society of America guidelines for healthcare-associated central nervous system infections [21].

### Pathophysiologic Cascade Model

Collectively, our data support a pathophysiologic cascade model rather than isolated events. Sustained preoperative and intraoperative intracranial hypertension increases the likelihood of hemorrhagic contusion expansion and severe postoperative swelling; severe swelling in turn compromises dural closure, predisposing to CSF leak; and CSF leak facilitates wound failure and CNS infection. This framework accounts for the observed overlap (46 events in 33 patients) and suggests that interventions targeting any single node—particularly ICP burden reduction or optimized dural reconstruction—may attenuate multiple downstream complications. While adult series describe similar overlapping morbidity after DC [9], the coupling between nodes appears tighter in children because of accelerated edema kinetics and developmental cranial compliance [11,12].

### Clinical Implications for Neurocritical Care and Operative Strategy

These observations carry direct implications for pediatric neurocritical care and operative strategy. Contemporary pediatric TBI guidelines emphasize early escalation for refractory intracranial hypertension [3]; our findings reinforce that permitting prolonged ICP elevation before DC may exact a postoperative cost in hemorrhagic progression and wound morbidity. Adult timing debates shaped by DECRA and RESCUEicp cannot be directly translated [5,6], yet pediatric observational data increasingly highlight heterogeneity and the need for individualized, physiology-guided decision-making [22,23]. From a technical standpoint, the complication profile supports meticulous pial and dural hemostasis, large basal decompression to minimize venous outflow obstruction, and the most watertight dural reconstruction feasible without constricting edematous tissue—potentially augmented by sealants or substitutes when indicated [7,9,26,27]. Patients with extreme intraoperative ICP, prolonged ICP burden, diffuse edema on imaging, or high systemic injury severity warrant intensified early postoperative surveillance with serial imaging and clinical monitoring for hemorrhagic progression or CSF egress.

### Limitations

This study is limited by its retrospective, single-center design and modest sample size, which restrict generalizability and causal inference. Operative details (dural substitute selection, sealant use, closure technique) and postoperative protocols, although standardized within our institution, vary across centers and may influence complication rates and overlap patterns. Our operational definition of CSF leak focused on clinically apparent wound liquorrhea or subgaleal collections; future studies should further distinguish radiographic from clinically consequential fistulae. Pediatric recovery is inherently developmental, and longer-term functional outcomes benefit from age-appropriate scales beyond conventional Glasgow Outcome Scale categorizations [28].

### Future Directions

Prospective multicenter pediatric cohorts with standardized definitions of complications, granular ICP “dose” phenotyping (cumulative burden, waveform analysis), and detailed reporting of closure techniques and intraoperative swelling severity are urgently needed. Such datasets will clarify whether earlier DC at lower ICP thresholds or protocolized high-swelling closure pathways can reduce hemorrhagic progression and CSF leak–driven infection without compromising decompressive efficacy [3,11,23].

## Conclusion

Decompressive craniectomy remains a life-saving intervention for refractory intracranial hypertension in pediatric severe traumatic brain injury, yet the early postoperative period carries a substantial neurosurgical complication burden. In our cohort, hemorrhagic complications predominated (34.9%), accounting for the majority of early morbidity—primarily expansion of pre-existing hemorrhagic contusions. Infectious complications (meningitis/ventriculitis) were strongly correlated with CSF leakage, which most often resulted from non-hermetic duraplasty performed under severe intraoperative brain swelling and was frequently accompanied by wound dehiscence and suture failure.

The risk of these early complications was closely associated with high intraoperative ICP (>35–40 mmHg), prolonged duration of refractory intracranial hypertension before surgery, and greater severity of primary brain injury, including diffuse axonal injury and concomitant systemic trauma. These factors appear to initiate a predictable cascade linking sustained ICP elevation, hemorrhagic progression, technical challenges in dural closure, and downstream wound/infectious sequelae.

Early, well-planned decompressive craniectomy—performed before extreme ICP accumulation—combined with meticulous hemostasis, generous yet as watertight as possible duraplasty (augmented by sealants or substitutes when indicated), and vigilant postoperative monitoring for CSF leakage and wound integrity may reduce complication incidence and severity, thereby improving neurological outcomes in children with severe traumatic brain injury. Multicenter prospective studies with standardized protocols are needed to confirm these observations and optimize timing and technique.

## Figures and Tables

**Figure 1.** Distribution of initial CT Marshall classification types among 83 pediatric patients with severe traumatic brain injury undergoing decompressive craniectomy. Type 3 and 4 lesions predominated (54.2% and 28.0%, respectively), while type 2 lesions occurred in 18.1% of patients. Abbreviations: CT, computed tomography.

**Table 1.** Early postoperative neurosurgical complications after decompressive craniectomy (N = 83) (Categories are not mutually exclusive.)

Complication category	n (%)	95% CI
Any early postoperative neurosurgical complication	33 (39.8)	29.2–50.4
Secondary intracranial hemorrhage	29 (34.9)	24.7–45.1
CNS infection (meningitis/ventriculitis)	7 (8.4)	2.4–14.4
Postoperative suture dehiscence	6 (7.2)	1.6–12.8
CSF leak (wound liquorrhea/subgaleal CSF collection)	4 (4.8)	0.2–9.4

**Table 2.** Baseline characteristics and ICP parameters by complication status

Characteristic	Group 1: No complications n = 50	Group 2: ≥1 complication n = 33	p-value
Age (years), mean ± SD	10.2 ± 4.1	9.8 ± 3.9	0.62
Male sex, n (%)	32 (64.0)	22 (66.7)	0.81
GCS at admission, mean ± SD	7.3 ± 1.0	4.6 ± 1.8	<0.001
ISS, mean ± SD	25.4 ± 6.2	35.8 ± 7.1	<0.001
CT-Marshall score, mean ± SD	2.8 ± 0.7	3.2 ± 0.5	0.01
Firsching classification, mean ± SD	1.4 ± 0.6	2.0 ± 0.7	0.002
Prehospital hypotension, n (%)	4 (8.0)	7 (20.0)	0.04
Prehospital hypoxia, n (%)	3 (6.0)	6 (17.0)	0.03
Intraoperative ICP (mm Hg), mean ± SD	28.6 ± 4.3	40.5 ± 13.5	0.0058
Duration ICP > 20 mm Hg (h), mean ± SD	1.6 ± 2.5	4.1 ± 5.9	0.0296

## Data Availability

The raw data supporting the conclusions of this article will be made available by the authors without undue reservation.

## References

[1] Jagannathan J, Okonkwo DO, Dumont AS, Outcome following decompressive craniectomy in children with severe traumatic brain injury: a 10-year single-center experience with long-term follow-up. J Neurosurg Pediatr. 2007;106(4 Suppl):268–275. doi:10.3171/ped.2007.106.4.268 17465359

[2] Pérez Suárez E, Serrano González A, Pérez Díaz C, Decompressive craniectomy in 14 children with severe head injury: clinical results with long-term follow-up and review of the literature. J Trauma. 2011;71(1):133–140. doi:10.1097/TA.0b013e318211071f 21818021

[3] Kochanek PM, Tasker RC, Carney N, Guidelines for the Management of Pediatric Severe Traumatic Brain Injury, Third Edition: Update of the Brain Trauma Foundation Guidelines. Pediatr Crit Care Med. 2019;20(3 Suppl 1):S1–S82. doi:10.1097/PCC.00001735 30829890

[4] Carney N, Totten AM, O’Reilly C, Guidelines for the Management of Severe Traumatic Brain Injury, Fourth Edition. Neurosurgery. 2017;80(1):6–15. doi:10.1227/NEU.00001432 27654000

[5] Cooper DJ, Rosenfeld JV, Murray L, ; DECRA Trial Investigators. Decompressive craniectomy in diffuse traumatic brain injury. N Engl J Med. 2011;364(16):1493–1502. doi:10.1056/NEJMoa1102077 21434843

[6] Hutchinson PJ, Kolias AG, Timofeev IS, ; RESCUEicp Trial Collaborators. Trial of decompressive craniectomy for traumatic intracranial hypertension. N Engl J Med. 2016;375(12):1119–1130. doi:10.1056/NEJMoa1605215 27602507

[7] Yang XF, Wen L, Shen F, Surgical complications secondary to decompressive craniectomy in patients with a head injury: a series of 108 consecutive cases. Acta Neurochir (Wien). 2008;150(12):1241–1247. doi:10.1007/s00701-008-0145-9 19005615

[8] Flint AC, Manley GT, Gean AD, Hemphill JC 3rd, Rosenthal G. Post-operative expansion of hemorrhagic contusions after unilateral decompressive hemicraniectomy in severe traumatic brain injury. J Neurotrauma. 2008;25(5):503–512. doi:10.1089/neu.2007.0442 18346002

[9] Stiver SI. Complications of decompressive craniectomy for traumatic brain injury. Neurosurg Focus. 2009;26(6):E7. doi:10.3171/2009.4.FOCUS0965 19485720

[10] Ban SP, Son YJ, Yang HJ, Chung YS, Lee SH, Han DH. Analysis of complications following decompressive craniectomy for traumatic brain injury. J Korean Neurosurg Soc. 2010;48(3):244–250. doi:10.3340/jkns.2010.48.3.244 21082053 PMC2966727

[11] Ardissino M, Tangney J, Ihugba J, Decompressive craniectomy in paediatric traumatic brain injury: a systematic review of current evidence. Childs Nerv Syst. 2019;35(2):209–216. doi:10.1007/s00381-018-3977-5 30215120 PMC6351512

[12] Manfiotto M, Beccaria K, Rolland A, Decompressive craniectomy in children with severe traumatic brain injury: a multicenter retrospective study and literature review. World Neurosurg. 2019;129:e56–e62. doi:10.1016/j.wneu.2019.04.215 31054345

[13] Ballestero MFM, Furlanetti LL, Augusto LP, Decompressive craniectomy for severe traumatic brain injury in children: analysis of long-term neuropsychological impairment and review of the literature. Childs Nerv Syst. 2019;35(9):1507–1515. doi:10.1007/s00381-019-04218-x 31264065

[14] von Elm E, Altman DG, Egger M, The Strengthening the Reporting of Observational Studies in Epidemiology (STROBE) statement: guidelines for reporting observational studies. Ann Intern Med. 2007;147(8):573–577. doi:10.7326/0003-4819-147-8-200710160-00010 17938396

[15] World Medical Association. World Medical Association Declaration of Helsinki: ethical principles for medical research involving human subjects. JAMA. 2013;310(20):2191–2194. doi:10.1001/jama.2013.281053 24141714

[16] Teasdale G, Jennett B. Assessment of coma and impaired consciousness. A practical scale. Lancet. 1974;2(7872):81–84. doi:10.1016/S0140-6736(74)91639-0 4136544

[17] Baker SP, O’Neill B, Haddon W Jr, Long WB. The injury severity score: a method for describing patients with multiple injuries and evaluating emergency care. J Trauma. 1974;14(3):187–196. 4814394

[18] Marshall LF, Marshall SB, Klauber MR, A new classification of head injury based on computerized tomography. J Neurosurg. 1991;75(Suppl):S14–S20. doi:10.3171/sup.1991.75.1s.0s14

[19] Firsching R, Woischneck D, Klein S, Classification of severe head injury based on magnetic resonance imaging. Acta Neurochir (Wien). 2001;143(3):263–271. doi:10.1007/s007010170106 11460914

[20] Jennett B, Bond M. Assessment of outcome after severe brain damage. Lancet. 1975;1(7905):480–484. doi:10.1016/S0140-6736(75)92830-5 46957

[21] Tunkel AR, Hasbun R, Bhimraj A, 2017 Infectious Diseases Society of America’s Clinical Practice Guidelines for Healthcare-Associated Ventriculitis and Meningitis. Clin Infect Dis. 2017;64(6):e34–e65. doi:10.1093/cid/ciw861 28203777 PMC5848239

[22] Xu J, Zhou R, Li J, The short-term impact of decompressive craniectomy in pediatric patients with severe traumatic brain injury: a retrospective matched cohort study. Children (Basel). 2025;12(10):1374. doi:10.3390/children12101374 41153556 PMC12562694

[23] Jaradat A, Al Barbarawi MM, Jamous M, Early versus late decompressive craniectomy in pediatrics with traumatic brain injuries: a retrospective study. World Neurosurg. 2025;196:123827. doi:10.1016/j.wneu.2025.123827 40010604

[24] Laws JC, Kochanek PM, Wolf MS. The path from intracranial pressure to clinical outcomes in pediatric traumatic brain injury. JAMA Netw Open. 2025;8(3):e250446. doi:10.1001/jamanetworkopen.2025.0446 40067307

[25] Marmarou A, Anderson RL, Ward JD, Impact of ICP instability and hypotension on outcome in patients with severe head trauma. J Neurosurg. 1991;75(Suppl):S59–S66.

[26] Huang YH, Lee TC, Chen WF, Wang YM. Safety of the nonabsorbable dural substitute in decompressive craniectomy for severe traumatic brain injury. J Trauma. 2011;71(3):533–537. 21768912 10.1097/TA.0b013e318203208a

[27] Oladunjoye AO, Schrot RJ, Zwienenberg-Lee M, Decompressive craniectomy using gelatin film and future bone flap replacement. J Neurosurg. 2013;118(4):776–782. 23394343 10.3171/2013.1.JNS121475

[28] Beers SR, Wisniewski SR, Garcia-Filion P, Validity of a pediatric version of the Glasgow Outcome Scale-Extended. J Neurotrauma. 2012;29(6):1126–1139. 22220819 10.1089/neu.2011.2272 PMC3325553

